# Revisiting the Language of Glycoscience: Readers, Writers and Erasers in Carbohydrate Biochemistry

**DOI:** 10.1002/cbic.201900377

**Published:** 2019-11-04

**Authors:** Simone Dedola, Michael D. Rugen, Robert J. Young, Robert A. Field

**Affiliations:** ^1^ Iceni Diagnostics The Innovation Centre Norwich Research Park Norwich Norfolk NR4 7GJ UK; ^2^ Department of Biological Chemistry John Innes Centre Norwich Research Park Norwich Norfolk NR4 7UH UK; ^3^ Present address: Cobra Biologics, Science Park University of Keele Newcastle-under-Lyme Staffordshire ST5 5SP UK; ^4^ Medicinal Chemistry Medicines Research Centre GlaxoSmithKline Stevenage Hertfordshire SG1 2NY UK; ^5^ Present address: Department of Chemistry and Manchester Institute of Biotechnology University of Manchester Manchester M1 7DN UK

**Keywords:** carbohydrates, epigenetics, glycoscience vocabulary, glycosyl hydrolase, glycosylation, glycosyltransferase

## Abstract

The roles of carbohydrates in nature are many and varied. However, the lack of template encoding in glycoscience distances carbohydrate structure, and hence function, from gene sequence. This challenging situation is compounded by descriptors of carbohydrate structure and function that have tended to emphasise their complexity. Herein, we suggest that revising the language of glycoscience could make interdisciplinary discourse more accessible to all interested parties.

## Graphical abstract

**The lack of template‐encoding** in glycoscience disconnects the “glyco code” from direct gene sequence control. Thus carbohydrate biosynthesis and glycan function depend upon a series of protein–carbohydrate interaction events. Descriptions of these events have tended to emphasise their complexity: here we suggest that revising the language of glycoscience could make interdisciplinary discourse more accessible to all parties.

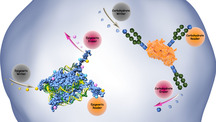

The perception that glycoscience—the chemistry and biology of carbohydrates—is both complex and ubiquitous in nature^1^, ^2^ has led to the notion that “*carbohydrates in molecular biology are like dark matter in the universe… poorly studied yet crucial to a full understanding of how things actually work*”.^3^ In contrast to nucleic acids and proteins (DNA codes for RNA codes for protein), the lack of template‐encoding disconnects the “glyco code”^4^ from direct gene sequence control. This results in carbohydrate biosynthesis and the biological function of glycans being dependent upon a series of protein–carbohydrate interaction events. Overall, the concerted actions of lectins, glycosyltransferases and/or glycoside hydrolases achieve the integrity of mature bioactive glycan structures. The intricacies of this landscape are made worse by the tendency of the glycoscience community to emphasise the complexities of the field, perhaps making it less accessible to the casual reader—the informed non‐expert—than it needs to be. The glycoscience community are not alone in this shortcoming, as highlighted by a Comment in *Nature* that suggests that *“Antibiotic resistance has a language problem. A failure to use words clearly undermines the global response to antimicrobials′ waning usefulness”*.^*[*5]^ Technological^6^ and informatics^7^ advances in glycoscience, alongside combinations of the two,^8^ are providing new ways to cut through the complexity, whilst comprehensive books of glycobiology topics provide entries in to the field.^9^ The introduction of stylized symbol nomenclature for glycans (SNFG; Figure 1) also represents an important step towards simplifying communication within and between interested disciplines^10^ along with guidelines for experimental design and data curation,^11^ and a repository for glycan structures.^12^

**Figure 1:**
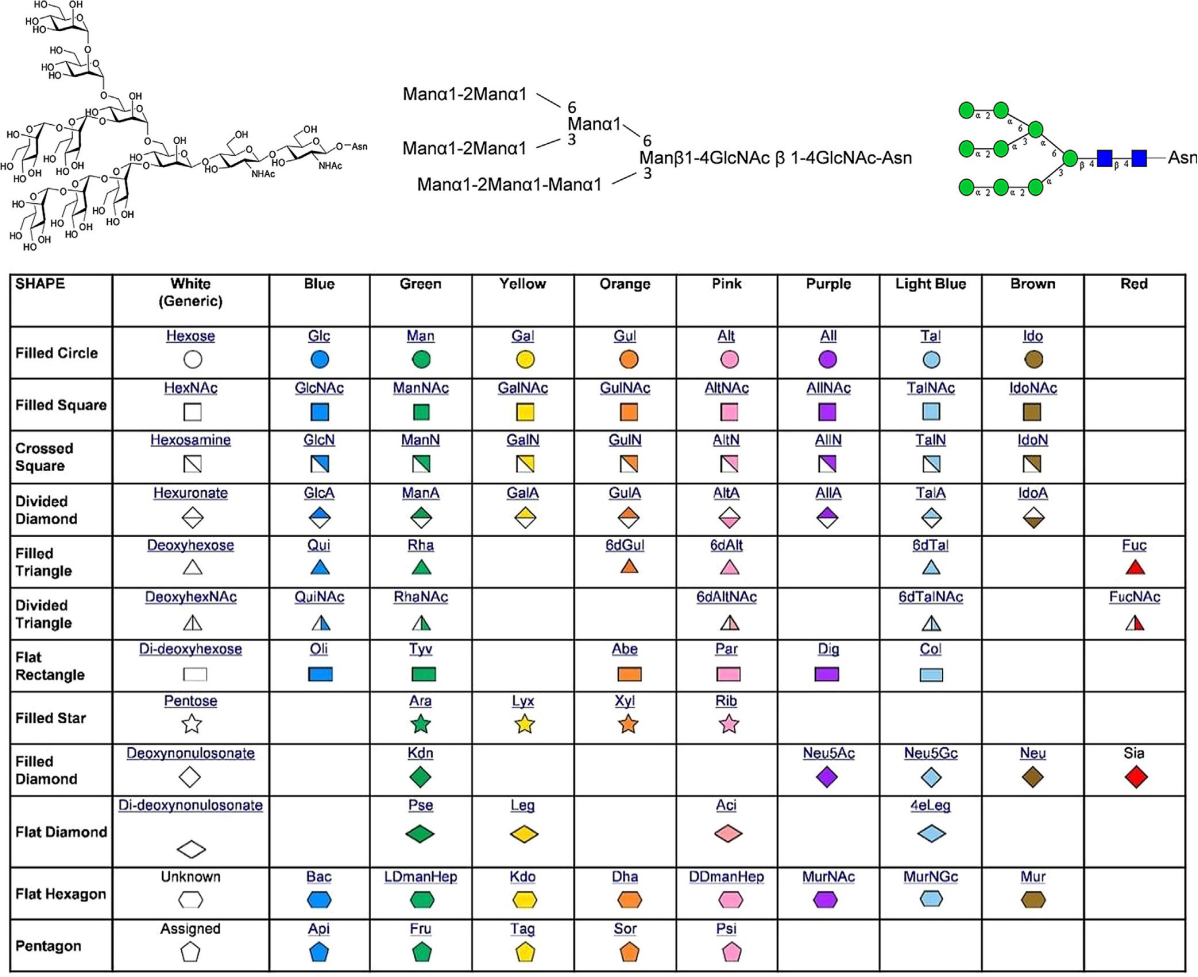
Representing glycan structures: simplification and standardization with stylized SNFG. Taken from ref. 10.

As discussed recently by Gabius,^13^ there are notable parallels between aspects of glycobiology and the epigenetic regulation of chromatin structure and function. The latter processes, which occur with exquisite precision, are typically referred to in stripped‐down terms as a series of *read*, *write* and *erase* events, making the field immediately accessible to outsiders. Indeed, this approach emulates computer programming's create, read, update and delete (CRUD)^14^—the four basic functions employed for persistent data storage.^15^ Herein, we consider the potential to recapitulate glycoscience language in the terms of epigenetic vocabulary.

In simple terms, epigenetics concerns small chemical changes (marks) in the chemical structure of chromatin—typically the histone proteins that organize and package DNA in chromosomes.^16^ Dynamic changes in these epigenetic protein marks impact on the physical accessibility of gene sequences for expression, rather than on the alteration of the genetic code per se. The profound biological consequences of these processes have attracted enormous attention over the past decade, given their central role in life and their disruption in disease.^17^ The molecular hallmarks of epigenetic regulation comprise a dynamic series of enzymatic modification steps that introduce or remove marks to the histone protein structure. Epigenetic writers, which introduce epigenetic marks on amino acid residues of the histones, include histone acetyltransferases (HATs, which N‐acetylate lysine), histone methyltransferases (HMTs, which N‐methylate lysine), protein arginine methyltransferases (PRMTs) and protein kinases (which O‐phosphorylate serine/threonine), amongst others. Epigenetic readers, which bind to epigenetic marks and amplify their impact on DNA packaging and hence gene accessibility for expression, include proteins containing bromodomains, chromodomains and Tudor domains. Epigenetic erasers, such as histone deacetylases (HDACs), lysine demethylases (KDMs) and phosphatases, catalyse the removal of epigenetic marks (Figure 2).

**Figure 2:**
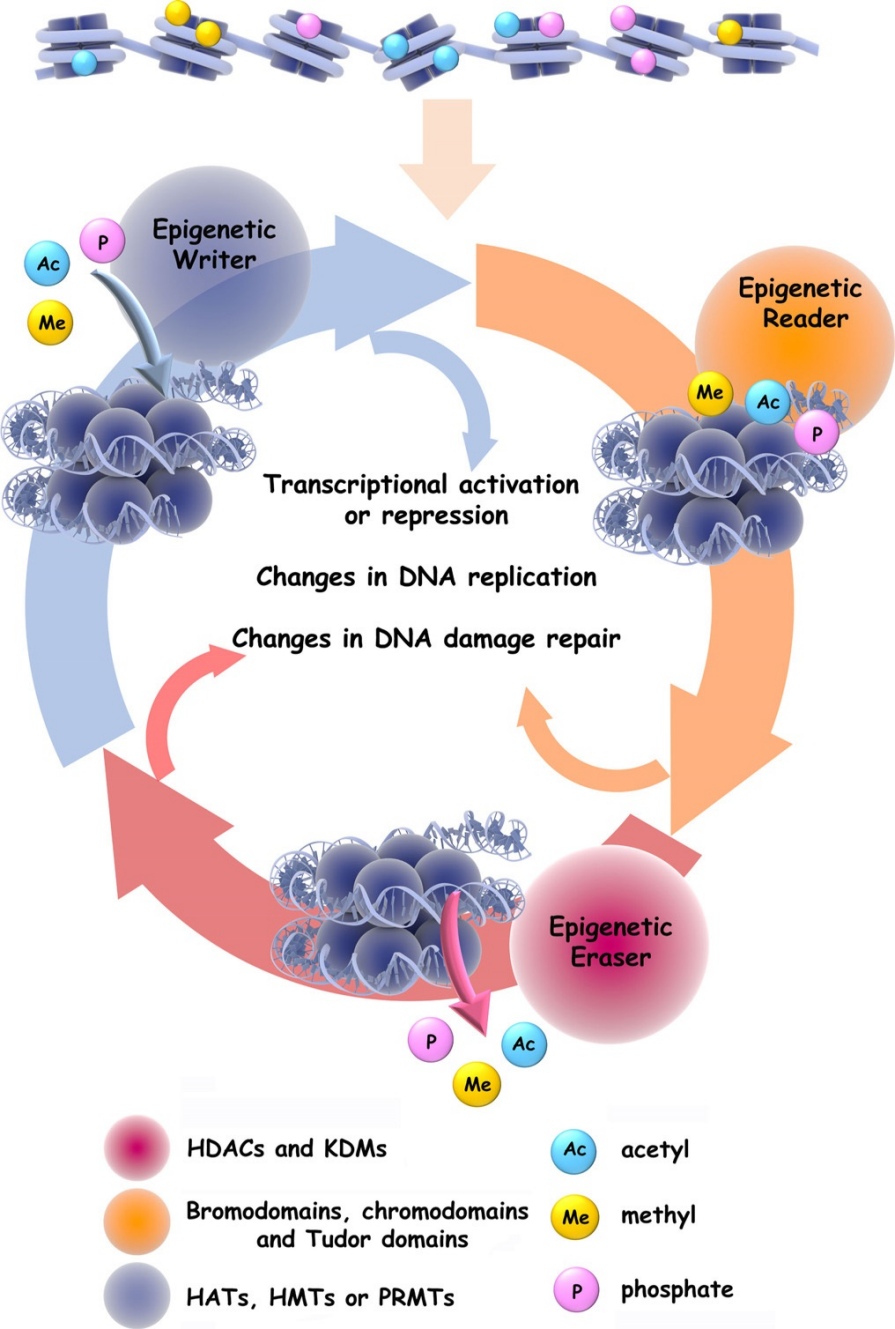
Epigenetic writers, readers and erasers. DNA packaged around histones gives a condensed genomic information package (top) that can be selectively unwound by epigenetic modification (e.g., acetylation, methylation of phosphorylation) to expose genes for transcription (turn on). Abbreviations used are given in the text. Adapted from ref. 17a.

The impact of the lysine N‐acetylation epigenetic mark is perhaps simplest to appreciate. Writing this mark results in the loss of a positive charge on the lysine side chain of a histone, thus removing the potential for interaction with the negatively charged DNA backbone and causing loosening the DNA–histone complex. The resulting opening up of the chromosome structure enables the localized activation (turning on) of gene expression. In the opposite sense, erasing a lysine acetylation mark drives a tighter assembly of the histone–DNA complex and silencing (turning off) gene expression.

The general principle of readers, writers and erasers prompts consideration of potential parallels between epigenetics and the control of glycan biosynthesis, structure and function. That is, does the notion of lectin readers, glycosyltransferase writers and glycosyl hydrolase erasers ring true in glycobiology? A convenient segue from epigenetics into glycoscience is provided by the reversible O‐GlcNAc modification of Ser/Thr residues in proteins.^18^ This central metabolic “rheostat”^19^ comprises a nutrient status‐responsive, post‐translational modification that impacts on protein–protein and protein–nucleic acid interactions. In turn regulating of cellular events including transcription and signal transduction, with implications in diabetes, Alzheimer's disease and cancer.

So how does the *O*‐GlcNAc cycle work? *O*‐GlcNAc transferase (OGT) writes and *O*‐GlcNAcase erases, providing a simple and reversible modification cycle that is orthogonal to protein phosphorylation and which has far‐reaching physiological impact (Figure 3).^20^

**Figure 3:**
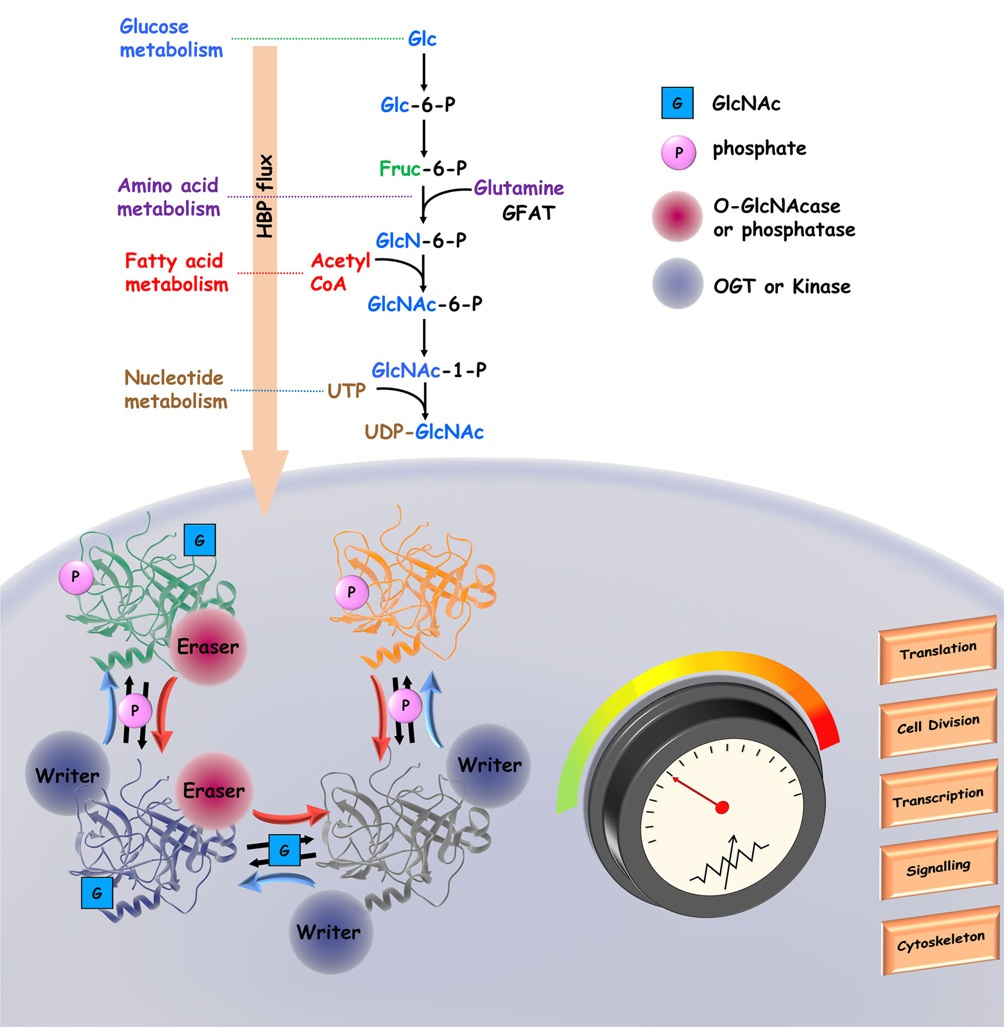
The *O*‐GlcNAc cycle and its impact on the modulation of cellular processes. Adapted from ref. 19.

In addition to glycosyltransferase writers and glycosyl hydrolase erasers, there are also potential readers in glycoscience—a function performed by lectins^21^ and the carbohydrate‐binding modules (CBMs)^22^ in multidomain CAZymes. The full read, write, erase combination in glycoscience is most easily exemplified by the proofreading and editing cycle associated with N‐linked glycoprotein biosynthesis. These processes are essential to ensuring the correct integrity and dynamics of cell‐surface glycoproteins, which contribute to the glycocalyx that dominates cell–cell interactions in the maintenance of healthy tissue and which underpin sperm–egg interactions during fertilisation, but which also serve as cellular receptors for a wide range of microbial pathogens.^9^

Asparagine‐linked protein N‐glycosylation starts in the endoplasmic reticulum, whereas the peptide chain is unfolded, and proceeds through protein folding to the Golgi apparatus, where the glycan components are processed to a mature state. This requires a highly organised distribution of processing machinery to achieve the fidelity and quality control needed to ensure biological function.^23^ Approximately 80 % of the proteins entering the secretory pathway are glycosylated in the ER and most of the proteins assembled in the ER feature N‐linked oligosaccharides. Most of the glycoproteins featuring mature *N*‐glycans are, as described by Aebi, “precisely heterogeneous” in their carbohydrate composition—a result of kinetically controlled processing.^24^ Nonetheless, to reach their final mature and bioactive form, in the early stage of biosynthesis all N‐linked glycoprotein are homogeneously glycosylated. This is a result of a precise lectin chaperone (reader) based proofreading mechanism in the ER, which discriminates between correctly folded and misfolded glycoproteins (Figure 4).^25^ Here the oligosaccharide plays a key role in presenting each glycoprotein for scrutiny by the sophisticated biological checkpoint process, which is referred to as glycoprotein quality control.^26^ This process ensures that only correctly folded glycoproteins are transported to the Golgi for further glycan processing in to mature glycoproteins. Unfolded and misfolded glycoproteins are retained in the ER for further folding attempts and are eventually degraded if the correctly folded status is not achieved.

**Figure 4:**
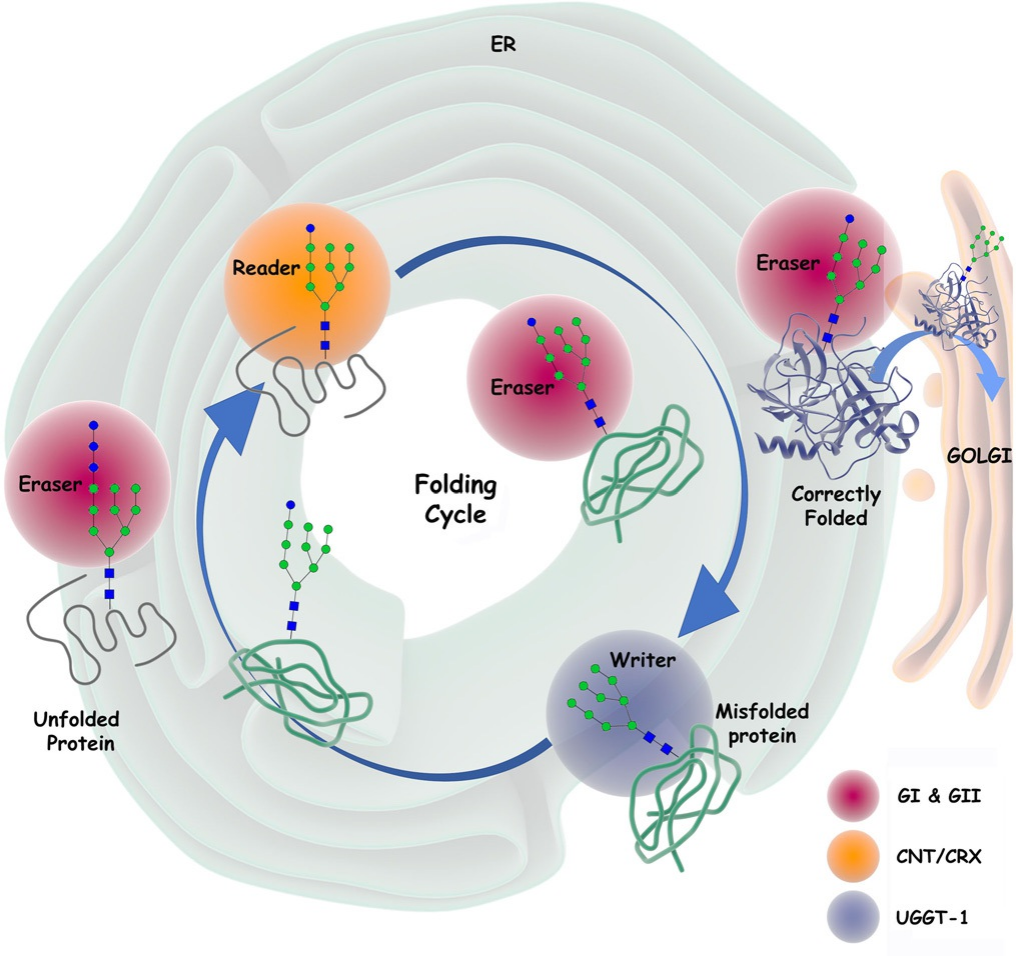
Carbohydrate writers, readers and erasers oversee the quality control of glycoprotein folding in the ER by modification of then‐linked glycan high mannose oligosaccharide core structure.

The glycoprotein quality control system presents clear parallels to the read, write and erase processes of epigenetic regulation. In the first step of glycosylation, Glc_3_Man_9_GlcNAc_2_ is transferred en bloc from an oligosaccharyl dolichol diphosphate to the nitrogen of an asparagine side chain in the nascent polypeptide chain by the writer oligosaccharyltransferase (OST).^27^ Immediately after the Glc_3_Man_9_GlcNAc_2_ is transferred, the eraser glucosidase‐I^28^ cleaves off the terminal glucose (Glc) residue, which is necessary to prevent the glycoprotein product rebinding to the OST. Subsequently, the eraser glucosidase‐II^29^ catalyses cleavage of a second glucose residue and the resulting monoglucosylated polypeptide is promptly sequestered by the calnexin (CNX)^30^ and calreticulin (CRT)^31^ lectin chaperone^32^ readers. These chaperones prevent aggregation of the unfolded glycopolypeptide chains, and assist in their correct folding by presentation to the oxidoreductase ERp57, which is responsible for effecting correct disulfide bond formation.^33^ Once the folded glycoprotein is released from the lectin chaperones readers, the eraser glucosidase‐II removes the final glucose residue and the glycoprotein undergoes inspection by the UDP‐glucose:glycoprotein glycosyltransferase (UGGT)^29^ (Figure 4).

If the correct glycoprotein folding is not accomplished, UGGT serves as a writer and re‐glucosylates the misfolded glycoprotein in preparation for recycling to the chaperones/ERp57 machinery^34^—the so‐called calnexin/calreticulin cycle (Figure 4).^35^ Following repeated failed folding attempts, the glycoprotein is degraded by the endoplasmic reticulum associated degradation system (ERAD).^36^ If correct folding is achieved, the glycoprotein is transported into the Golgi apparatus for further processing of the glycan to provide the mature glycoprotein.

## Conclusion

It is widely recognised that carbohydrates play important roles in biological molecular recognition, and have a profound impact on human health and medicine. Nonetheless, there is merit in simplifying the language of glycoscience to make it more accessible to the uninitiated. In turn, this might facilitate a focus on the principles and implications of glycosylation in biology, rather than risking drowning in the detail of structural complexity. The notion of accessible vocabulary in glycoscience is not new: it was already evident in Hood, Huang and Dreyer's 1977^37^ description of differentiation antigens as cell‐surface “area codes”; and the potential of cell‐surface carbohydrates, lectins, enzymes and carbohydrate‐binding antibodies in Feizi's 1981^38^ “cellular addresses”, “postmen, policeman and traffic signs” “involved in the obedient interpretation of area codes”. Similar thoughts were explored in Brandley and Schnaar's 1986^39^ “potential carbohydrate ”language“ involved in intercellular interactions”, while Hakomori's 2002^40^ “glycosynapse”—microdomains of glycolipids—seeks to draw parallels to the “immune synapse” assembly that contributes to cell adhesion and signalling. As highlighted in the cross‐disciplinary article by Bertozzi and Kiessling in 2001,^41^ “chemical tools have proven indispensable for studies in glycobiology”. Perhaps it is time to revisit the terminology of glycoscience, to make interdisciplinary communication more straightforward and to support marketing and engagement beyond the immediate field. Reference to lectin readers, glycosyltransferase writers, and glycosyl hydrolase erasers could therefore be worth wider (re)consideration.

## Conflict of interest

*The authors declare no conflict of interest*.

## Supporting information

As a service to our authors and readers, this journal provides supporting information supplied by the authors. Such materials are peer reviewed and may be re‐organized for online delivery, but are not copy‐edited or typeset. Technical support issues arising from supporting information (other than missing files) should be addressed to the authors.

Supplementary
