# Supplementary material for: Revisiting the Language of Glycoscience: Readers, Writers and Erasers in Carbohydrate Biochemistry
Source: Chembiochem. 2019 Nov 4;21(3):423–7. doi: 10.1002/cbic.201900377 (PMC7463168; doi:10.1002/cbic.201900377)
Supplement: Supplementary file 1 — Supplementary [file CBIC-21-423-s001.pdf]

Author Contributions

S.D. Conceptualization: Equal; Writing - Original Draft: Equal; Writing - Review & Editing: Equal
M.R. Conceptualization: Equal; Writing - Original Draft: Equal; Writing - Review & Editing: Equal
R.Y. Conceptualization: Equal; Writing - Original Draft: Equal; Writing - Review & Editing: Equal
R.F. Conceptualization: Equal; Writing - Original Draft: Equal; Writing - Review & Editing: Equal.
